# Chemical residues in tomato plant tissue and *in vitro* toxicology profiles of Nemarioc-AL and Nemafric-BL phytonematicides on Raw 264.7 macrophage cells

**DOI:** 10.3389/fpls.2025.1681660

**Published:** 2026-02-05

**Authors:** Tshepo S. Mashela, Ashwell R. Ndhlala, Esam Elgorashi, Raymond T. Makola

**Affiliations:** 1 Green Biotechnology and Innovation, Department of Plant Production, Soil Science and Agricultural Engineering, University of Limpopo, Polokwane, South Africa; 2 Toxicology and Ethnoveterinary Medicine, Onderstepoort Veterinary Institute, Agricultural Research Council, Pretoria, South Africa; 3 Biochemistry, Microbiology and Technology Laboratory, Department of Biochemistry, University of Limpopo, Polokwane, South Africa

**Keywords:** antagonist, bioactive substance, cucurbitacin residues, cytotoxicity, necrosis, plant parasitic nematodes

## Abstract

A shift from synthetic chemical nematicides to bionematicides in plant protection has led to the development of Nemarioc-AL and Nemafric-BL phytonematicides to manage plant parasitic nematodes. However, there is a lack of information on the accumulation of cucurbitacin residues and the cytotoxicity of phytonematicides on non-target entities. The aims of the study were to determine the cucurbitacin residue accumulation after the application of Nemarioc-AL and Nemafric-BL phytonematicides in tomato fruits and to determine their cytotoxic effects on a eukaryotic (Raw 264.7 cell line) model system. Two separate trials for Nemarioc-AL phytonematicide and Nemafric-BL phytonematicide, each applied at 3%, were conducted concurrently on sandy loam, dark soil, red soil, silt soil, sandy soil, and sandy loam (+). Each trial was arranged in a randomized complete block design (RCBD) and replicated six times. 3-(4,5-Dimethylthiazol-2-yl)-2,5-diphenyl-2*H*-tetrazolium bromide (MTT) viability assay was used to assess the cytotoxicity of Nemarioc-AL and Nemafric-BL phytonematicides, and the Annexin-V and DAPI apoptosis assay was performed on Raw 264.7 macrophage cells. In Nemarioc-AL phytonematicide-treated soil type, the highest accumulation of cucurbitacin B residues in fruits was observed on sandy loam (+) (37.1 ng/g), followed by red soil with 27.0 ng/g and then sandy soil with 21.7 ng/g, and dark soil showed the least at 20.3 ng/g. The phytonematicides were non-toxic at lower concentrations, ≤1.25 mg/mL. However, the higher concentrations (>1.25 mg/mL) of phytonematicides exhibited cytotoxic effects on the Raw 264.7 cell line, with 50% cell viability in comparison with curcumin (100 μM). The IC_50_ values for Nemarioc-AL and Nemafric-BL phytonematicides on Raw 264.7 cell lines were 0.55 and 1.6 mg/mL, respectively. Similar to the MTT viability assay, the Annexin-V and DAPI apoptosis assay did show that the low concentrations of phytonematicides (0.313 mg/mL) had no signs of apoptosis or necrosis; however, high concentrations (10 mg/mL) had signs of apoptosis as opposed to necrosis. Therefore, the products can be used at lower concentrations to manage nematodes and avoid the toxicity of the products.

## 1 Introduction

Globally, a successful production of tomato (*Solanum lycopersicum* L.) plants infested by plant parasitic nematodes (PPNs) has relied heavily on synthetic chemical nematicides (SCNs) to obliterate this economic pest (Aguilar-Marcelino et al., 2023). Synthetic chemical nematicides are underscored by the accumulation of chemical residues in produce, toxicity to non-target organisms, the emergence of resistant strains, and environmental unfriendliness (Fenibo et al., 2021). As a result, Nemarioc-AL and Nemafric-BL phytonematicides were developed as bionematicides, an antagonist product against PPNs, from the fermented fruit extracts of indigenous plants wild cucumber (*Cucumis myriocarpus* Naud.) and wild watermelon (*Cucumis africanus* L.), respectively (Kristkova et al., 2003; Pelinganga, 2013; Mashela et al., 2017). Biopesticides suppress pests through multiple modes of action, while SCNs have a single active ingredient with prevalent pest resistance incidents reported (Mashela et al., 2015; Dube, 2016; Fenibo et al., 2021). Since these two antagonist products were developed and used for plant protection purposes, care should be taken regarding chemical residues in tomato fruits and cytotoxicity, such that humans and non-target entities are not compromised if exposed.

Sustainable Development Goals (SDGs) directly linked to the use of phytonematicides as biopesticides cover land farming, closing the global poverty gap, ending hunger, and improving nutrition (Hák et al., 2016; Fenibo et al., 2021, 2022). Despite biopesticide products showing promise as the future alternative to SCNs, which aligns with a context of SDGs, they currently constitute less than 5% in the agrochemical market (Sachdev and Singh, 2016; Soetopo and Alouw, 2023). The adoption of biopesticides in the agrochemical market is constrained by toxicity profiling and improper testing of the products, and information on safety to humans is not emphasized. The South African regulatory authority, “Fertilisers, Farm Feeds, Agricultural Remedies and Stock Remedies Act”, requires that any agricultural remedy undergo a rigorous registration process before use in compliance with Act 36 of 1947. As such, this act requires that detailed chemical residue and toxicological data from accredited laboratories be submitted prior to the registration of the products.

Cucurbitacins, as tetracyclic triterpenes, are classified into groups, A, B, C, D, E, etc., and mainly isolated from the Cucurbitaceae family (Kaushik et al., 2015; Delgado-Tiburcio et al., 2022; Varela et al., 2022). In Nemarioc-AL phytonematicide, the lead chemical compound (LCC) is cucurbitacin A (C_32_H_46_O_9_), which is a semipolar triterpenoid chemical compound molecule that is highly unstable and rapidly breaks down into leptodermin (C_27_H_38_O_8_) and cucumin (C_27_H_40_O_9_), which are bioactive against certain insects (Chen et al., 2005). In contrast, LCC in Nemafric-BL phytonematicide is cucurbitacin B (C_32_H_46_O_8_), which is a non-polar chemical compound molecule that is highly stable (Chen et al., 2005). The LCCs are the active ingredients or bioactive compounds of the phytonematicides, which have allelopathic effects and can inhibit the growth of other living entities (Šoln et al., 2022). The challenge of toxicity on tomato plants was resolved using the Curve-fitting Allelochemical Response Dose (CARD) algorithm computer-based model, previously developed in Australia (Liu et al., 2003), adopted by Mashela et al. (2017) to develop the Mean Concentration Stimulation Point (MCSP). The MCSP is the concentration that will suppress nematode numbers without inducing toxicity to the protected crops. However, there is a lack of information on the response of humans to the phytonematicides.

The cytotoxic profiles of Nemarioc-AL and Nemafric-BL phytonematicides were assessed using the 3-(4,5-dimethylthiazol-2-yl)-2,5-diphenyl-2*H*-tetrazolium bromide (MTT) assay, which measures cell viability. These phytonematicides may exhibit cytotoxic effects if they disrupt essential metabolic processes, leading to abnormal energy levels and ultimately cell death (Kamanja et al., 2018). Cytotoxicity refers to the capacity of a bioactive or synthetic compound to kill living cells (Celik, 2018). Cytotoxicity-inducing compounds like alkaloids, terpenoids, and phenolics are found in natural plant food as well as artificial compounds found in plant produce, medications, and environmental contaminants (León-Mejía et al., 2021). Therefore, if detected in unwanted situations, they are precisely called xenobiotics, which include botanical and synthetic chemicals, pesticides, azo dyes, phenolics, polycyclic aromatic hydrocarbons (PAHs), halogenated compounds, personal care products (PCPs), pharmaceutical active compounds (PhACs), nitroaromatic compounds, triazines, and chlorinated compounds (Godheja et al., 2016; Dhakal et al., 2018; Miglani et al., 2022). Hence, non-tested products can have negative effects on humans and non-target organisms if exposed to this broad range of foreign xenobiotic chemicals. Therefore, the objective of this study was i) to test whether Nemarioc-AL and Nemafric-BL phytonematicides would result in the accumulation of cucurbitacin residues in tomato fruits and ii) to determine whether Nemarioc-AL and Nemafric-BL phytonematicides would have cytotoxic effects on humans using the Raw 264.7 cell line model system.

## 2 Materials and method conditions

### 2.1 Greenhouse conditions

#### 2.1.1 Study site, research design, and cultural practices

The trials were conducted in a greenhouse at the Green Biotechnologies Research Centre of Excellence (GBRCE), University of Limpopo, South Africa (23°53″10′S, 29°44″15′E). This study was carried out during the autumn and winter of 2023 (March–August). Thermostatically activated fans and a wet wall were used to control the ambient temperatures of the greenhouse, which averaged 25°C during the day and 21°C at night. Two separate trials for Nemarioc-AL phytonematicide and Nemafric-BL phytonematicide were conducted concurrently; each phytonematicide was applied directly into sandy loam, dark soil, red soil, silt soil, sandy soil, and sandy loam (+) at 3%; arranged in a randomized complete block design (RCBD); and replicated six times. Nemarioc-AL phytonematicide and Nemafric-BL phytonematicide were first prepared using a locally developed method at the University of Limpopo, South Africa (Mashela et al., 2017). Tomato plant seeds (cv. ‘Floradate’) were planted in a 200-hole seedling tray with Hygromix-T (Hygrotech, Pretoria North, South Africa), set up on a greenhouse bench, and given frequent irrigation. Each soil was pasteurized and filled in 20-cm-diameter plastic pots with a total volume of 2.700 mL of growing mix. At the four-leaf stage, tomato seedlings were hardened off for 1 week and then transplanted directly into 20-cm plastic pots. Each plant received 5 g of 2:3:2 (26) NPK fertilization upon transplanting; 2 weeks later, 2 g of 2:1:2 (43) Multifeed (Nulandies, Johannesburg, South Africa) was applied. Monitoring for insect pests and diseases inside the greenhouse was carried out daily. Basil (*Ocimum basilicum* L.) and *Momordica* (*Momordica balsamina* L.) were used as repellent plants inside the greenhouse. At 150 days after the application of Nemarioc-AL and Nemafric-BL phytonematicides, shoots were cut off from the roots above the ground and weighed for fresh shoot and root mass. After that, they were dried in an oven at 52°C for 72 hrs and then ground in a Wiley mill.

#### 2.1.2 Determining soil pH and soil electrical conductivity

Soil pH and soil electrical conductivity (EC) were determined according to Okalebo et al. (2002). The method involves weighing 20 g of each soil sample and adding 50 mL of deionized water to a 100-mL tube. The mixture was stirred for 10 min using a shaker (Labcon, Johannesburg, South Africa) and allowed to settle for 30 min, and soil pH was measured by using Multiparameter EC Meter edge (Hanna Instruments (Pty) Ltd, Johannesburg, South Africa). Then, the mixture was allowed to settle for 1 hr, and the soil electrical conductivity in the supernatant liquid was measured.

#### 2.1.3 Determination of residues in different plant parts

The accumulation of cucurbitacin residues in tomato plant fruits, leaves, stems, and soil was carried out using ultra-high performance liquid chromatography–quadrupole time-of-flight mass spectrometry (UHPLC–QTOF–MS) as described in Mpai and Sivakumar (2020) and Mokgalabone et al. (2025). Approximately 0.25 g each of dried and ground roots, stems, leaves, and fruits was separately weighed out into a 15-mL Falcon tube and then extracted with 10 mL of methanol with vortexing and standing overnight. Following centrifugation at 14,000 rpm for 7 min, each of the samples was diluted 5×, and the supernatant was transferred into 1.5-mL vials for further analysis. A high-resolution Waters Synapt G2 QTOF mass spectrometer (MS) coupled to a Waters Acquity ultra-performance liquid chromatograph (UPLC) (Waters, Milford, MA, USA) was used to analyze the targeted metabolites (Waters, Milford, MA, USA). The Waters BEH C18 2.1 × 50 mm, 1.7-μm column was used to achieve separation. The compounds were measured in relation to a calibration curve that was created by injecting cucurbitacin A and cucurbitacin B standards in a range of 1 to 50 ng/mL.

### 2.2 *In vitro* condition

#### 2.2.1 Cell propagation, maintenance, and sample preparation

Each of the two samples of Nemarioc-AL phytonematicide and Nemafric-BL phytonematicide was serially diluted (0, 0.812, 1.625, 3.25, 6.5, 12.5, 25, 50, and 100 mg/mL) in a microplate well. Treatments were arranged in a completely randomized design (CRD), replicated three times. Curcumin was used as a standard at 100 μM in the current study. Raw 264.7 macrophage cells were bought from ATCC and stored in a −80°C freezer until required. The cell lines were thawed, suspended, and cultured in a Dulbecco’s Modified Eagle Medium (DMEM) supplemented with 1× Penicillin, streptomycin, and neomycin (PSN) and 10% fetal bovine serum (FBS) within a T75 culture flask. Cells were allowed to grow for a period of 1 week in a CO_2_ incubator at 37°C and 5% CO_2_. The medium was removed, and the remaining cells attached to the flask were detached using a cell scraper. Cell counting was performed using a 1:1 ratio of cell solution to trypan blue dye, and Countess (Thermo Fisher Scientific (Pty) Ltd, Johannesburg, South Africa) was used to count the cells.

#### 2.2.2 MTT viability assay

In order to examine the toxic levels of Nemarioc-AL and Nemafric-BL phytonematicides, 100 µL of the cell cultures was seeded in 96-well microplates at 5 × 10^5^ cells per well overnight and followed by treatment with various concentrations of Nemarioc-AL and Nemafric-BL phytonematicides for 24 hrs. After 24 hrs of treatment, just before the addition of 5 mg/mL of MTT dye, cell morphology pictures of Raw 264.7 macrophage cells were captured using Olympus CKX53, LC micro software (Makola et al., 2020). Thereafter, MTT was added to each well, and then the microplates were incubated at 37°C in 5% CO_2_ incubator for 2 hrs. The formed formazan products were dissolved in 2% HCl acidified isopropanol for 10 min. Thereafter, absorbance was measured using a GloMax-Multi microplate reader (Promega Corporation, Madison, Wisconsin, USA) at 560 nm. Percentage viability was calculated using the formula:


$$Cell\;viability\;\left(\%\right)=\frac{Average\;OD\;\left(experimental\;group\right)\;}{Average\;OD\;\left(untreated\;group\right)}\times 100$$

#### 2.2.3 Apoptosis assay

The apoptotic effects of Nemarioc-AL and Nemafric-BL phytonematicides on Raw 264.7 cells were assessed using Annexin-V FITC and DAPI kit (Abcam Inc., Cambridge, Massachusetts, USA) according to the manufacturer’s protocol. This assay kit detects apoptotic features, such as flipping of the plasma membrane inward out for exposing the phosphatidylserine, membrane integrity, and chromosomal condensation. Cells were seeded at a density of 2 × 10^5^ cells/mL on coverslips in six-well plates and incubated overnight. Cells were treated with 10 mg/mL, 0.313 mg/mL, and 100 μM curcumin for 24 hrs. This was followed by the removal of medium and washing of cells with 1× Phosphate Buffered Saline (PBS). Cells were stained with DAPI and Annexin-V for 20 min in the dark at room temperature (RT). Cells on the coverslips were fixed for 30 min with 3.7% paraformaldehyde. Coverslips were then mounted with 100% glycerol on microscope slides, and images were captured using Evos M3000 (Thermo Fisher, USA).

### 2.3 Data analysis

Data were subjected to two-way analysis of variance (ANOVA), and mean separation was performed using Duncan’s Multiple Range Test at a probability of 5%. Data for concentration response curves were analyzed using GraphPad Prism^®^ version 5.2 (GraphPad Software Inc., San Diego, CA, USA) (Motulsky, 2007). The IC_50_ values were generated using non-linear regression in GraphPad Prism (Lyles et al., 2008).

## 3 Results

### 3.1 Greenhouse condition

#### 3.1.1 Phytonematicides on plant tissues and soil parameters

The influence of Nemarioc-AL and Nemafric-BL phytonematicides in dark soil, red soil, silt soil, sandy soil, and sandy loam soil (+) grown tomato plants has significantly decreased the pH level of the selected soil types, except for sandy loam soil (**Tables 1**, **2**). A decrease in soil pH levels was observed in all the selected soil types when comparing the initial soil at transplanting and the final pH at termination of the experiment (**Tables 1**, **2**). In contrast, Nemarioc-AL phytonematicides increased soil electrical conductivity on the stated soil types (**Table 1**), while Nemafric-BL phytonematicides have shown an increase, a decrease, or no effect on soil electrical conductivity of different soil types (**Table 2**). However, soil texture and organic matter did not influence the accumulations of cucurbitacin residues in tomato plant parts (**Table 3**).

**Table 1:** The pH and EC for sandy loam, dark soil, red soil, silt soil, sandy soil, and sandy loam (+) during planting (IN) and after (FN) 150 days of application of Nemarioc-AL phytonematicide (n = 18).

Soil types	pH (IN)	pH (FN)	EC, dS/m (IN)	EC, dS/m (FN)
Sandy loam	7.12^ec^	7.10^b^	1.71^c^	1.89^c^
Dark soil	8.52^a^	7.59^a^	2.23^a^	2.30^a^
Red soil	7.93^ab^	5.82^c^	1.89^b^	2.11^ab^
Silt soil	7.66^ab^	5.32^d^	1.53^d^	1.84^cd^
Sandy soil	7.4^bc^	5.0^e^	1.32^e^	1.67^d^
Sandy loam (+)	7.01^c^	4.71^f^	0.91^f^	1.94^bc^

Means with different letters within each column show significant differences at p ≤ 0.01.

EC, electrical conductivity.

**Table 2:** The pH and EC for sandy loam, dark soil, red soil, silt soil, sandy soil, and sandy loam (+) during planting (IN) and after (FN) 150 days of application of Nemafric-BL phytonematicide (n = 18).

Soil types	pH (IN)	pH (FN)	EC, dS/m (IN)	EC, dS/m (FN)
Sandy loam	7.06^e^	7.05^b^	1.69^c^	1.74^c^
Dark soil	8.51^a^	7.26^a^	2.32^a^	2.35^a^
Red soil	7.91^b^	5.22^c^	1.87^b^	1.88^bc^
Silt soil	7.75^c^	4.57^e^	1.06^d^	1.96^b^
Sandy soil	7.37^d^	4.65^de^	1.94^b^	1.85^bc^
Sandy loam (+)	7.09^e^	4.83^d^	0.90^e^	1.95^b^

Means with different letters within each column show significant differences at p ≤ 0.01.

EC, electrical conductivity.

**Table 3:** Soil texture and organic matter content for sandy loam, dark soil, red soil, silt soil, sandy soil, and sandy loam (+) during planting.

Soil types	Soil texture			Organic matter (%)	Reference
	Sand (%)	Clay (%)	Silt (%)		
Sandy loam	65	30	5	1–6	Ramputla, 2019
Dark soil	22	40	38	5–12	Dube, 2016
Red soil	65	7	28	0.5–3	Kumar et al., 2013
Silt soil	8	12	80	2–6	Okalebo et al., 2002
Sandy soil	89	6	5	0.5–2	Dube, 2016
Sandy loam (+)	65	30	5	1–6	Ramputla, 2019

**Figure 1:**
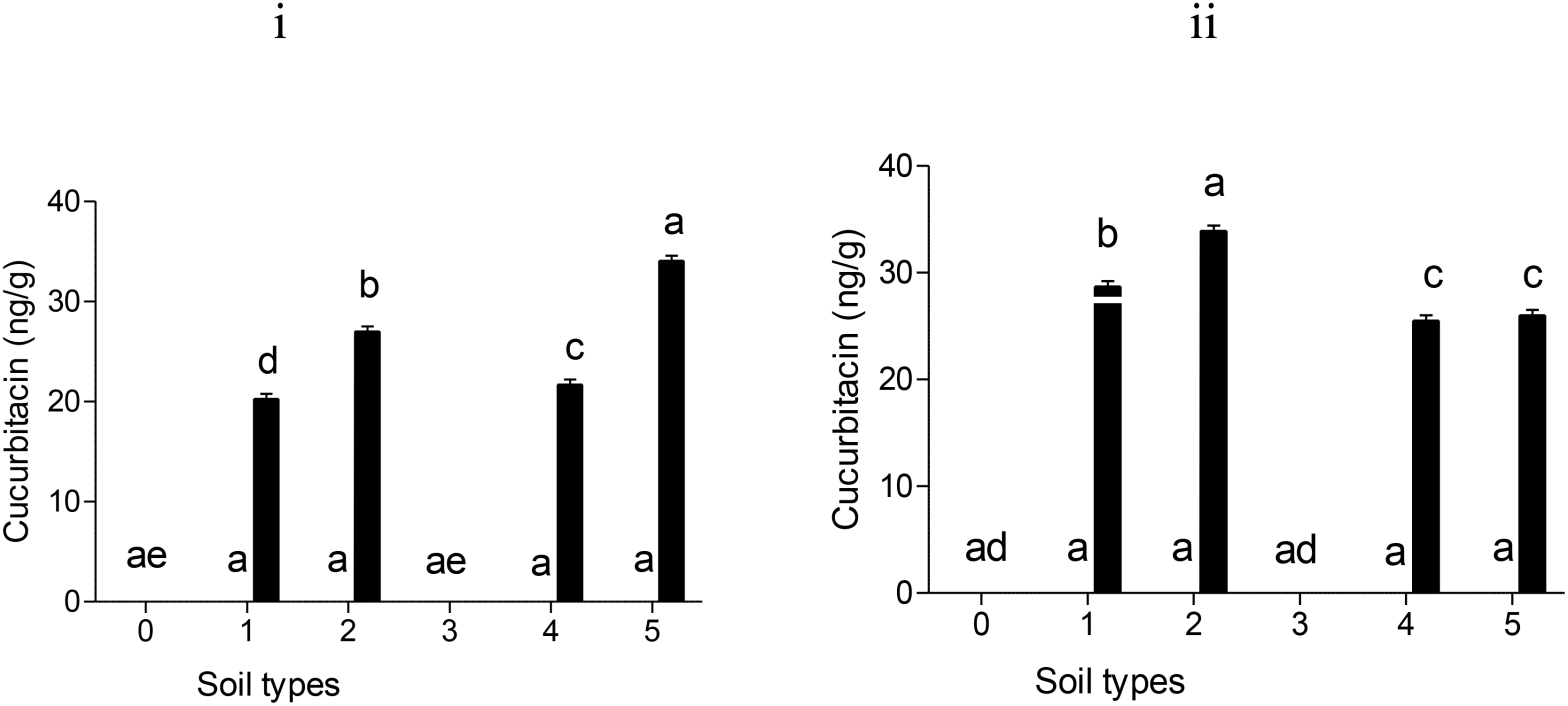
Levels of cucurbitacin A (□) and cucurbitacin B (▪) residues detected in tomato fruits: (i) Nemarioc-AL and (ii) Nemafric-BL phytonematicide. The plants were grown under different soil types (0) sandy loam, (1) dark soil, (2) red soil, (3) silt soil, (4) sandy soil, and (5) sandy loam (+) (n = 36). Bars ( ± SE) with different letters showing significant differences between treatments at p ≤ 0.01.

Residues of cucurbitacin B were detected in tomato fruits treated with Nemarioc-AL and Nemafric-BL phytonematicides after 150 days of the application of the products on sandy loam, dark soil, red soil, sandy soil, and sandy loam soil (+) (**Figures 1i, ii**). In contrast, silt soil did not produce mature fruits fit to be analyzed. In Nemarioc-AL phytonematicide-treated soil type, the highest levels of cucurbitacin B were detected in sandy loam (+) (34.1 ng/g), followed by red soil with 27.0 ng/g, then sandy soil with 21.7 ng/g, and lastly dark soil with 20.0 ng/g (**Figure 1i**). In Nemafric-BL phytonematicide, the highest concentration of cucurbitacin B residues in fruits was detected in red soil with 33.9 ng/g, followed by dark soil with 28.7 ng/g, then sandy loam (+) with 26.0 ng/g, and lastly sandy soil with 25.5 ng/g (**Figure 1ii**). No accumulation of cucurbitacin A was detected in fruits treated with Nemarioc-AL and Nemafric-BL phytonematicides (**Figure 1i, ii**).

**Figure 2:**
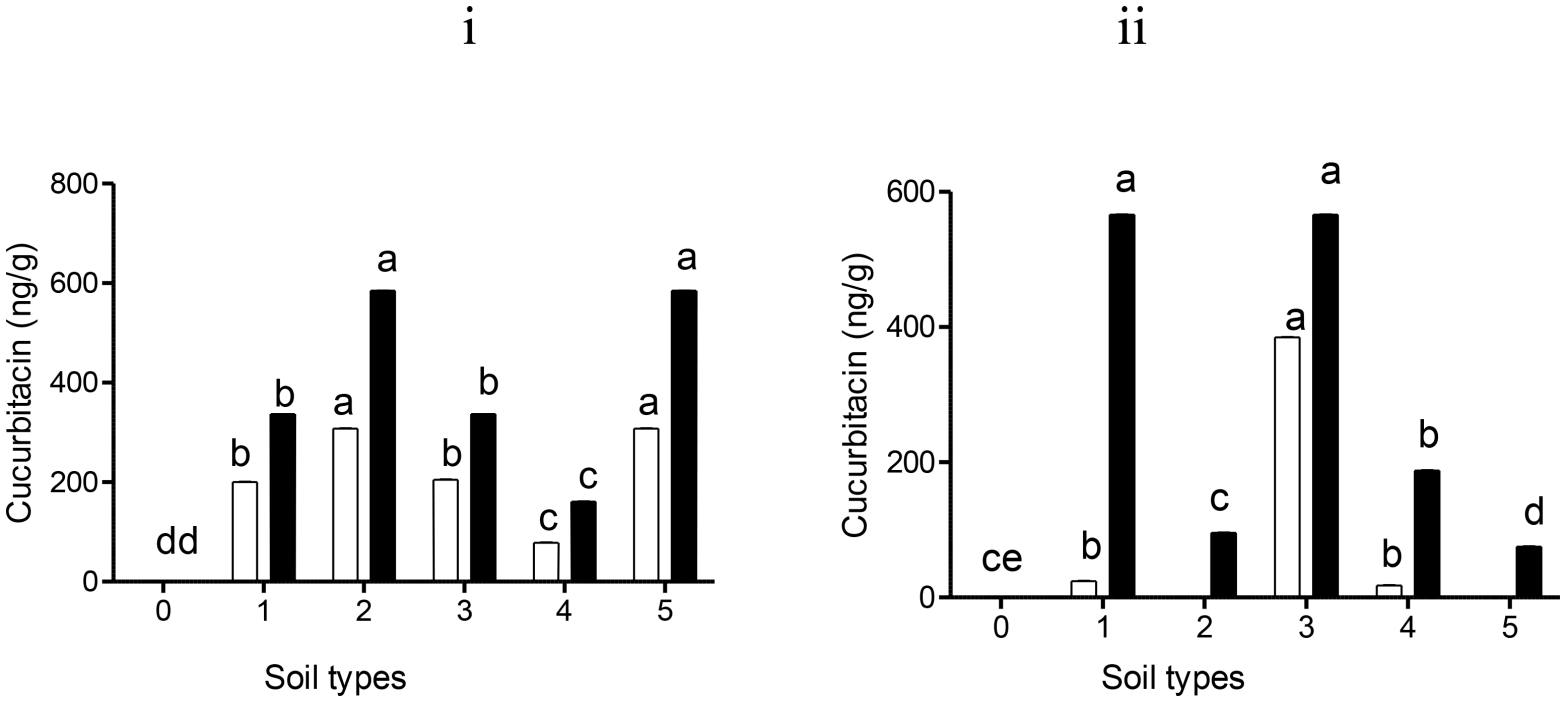
Levels of cucurbitacin A (□) and cucurbitacin B (▪) residues detected in tomato leaves: (i) Nemarioc-AL and (ii) Nemafric-BL phytonematicide. The plants were grown under different soil types (0) sandy loam, (1) dark soil, (2) red soil, (3) silt soil, (4) sandy soil, and (5) sandy loam (+) (n = 36). Bars ( ± SE) with different letters showing significant differences between treatments at p ≤ 0.01.

**Figures 2i and ii** shows tomato plants grown on different soil types treated with Nemarioc-AL and Nemafric-BL phytonematicide. The accumulation of both cucurbitacin A and cucurbitacin B was detected. Leaves harvested from red soil and sandy loam (+) (307.7 ng/g) applied with Nemarioc-AL phytonematicide attained high concentrations of cucurbitacin A residues, with silt soil (250.1 ng/g), dark soil (200.5 ng/g), and sandy soil (78.3 ng/g) (**Figure 2i**). Cucurbitacin B accumulation increased in leaves harvested on red soil and sandy loam (+) (584.8 ng/g), having a non-significant difference from each other, with dark soil and silt soil (336.7 ng/g) following a similar pattern; sandy soil showed the least (161 ng/g) (**Figure 2i**). In contrast, in leaves treated with Nemafric-BL phytonematicide, cucurbitacin B residues was at its peak from both dark soil and silt soil (566.2 ng/g), with a decrease on sandy soil (187.6 ng/g) and red soil (95.8 ng/g), and sandy loam (+) (75.2 ng/g) showed the least (**Figure 2ii**). A maximum of cucurbitacin A was detected in silt soil (384.6 ng/g), dark soil (24 ng/g), and sandy soil (17.8 ng/g); no traces of cucurbitacin A were detected in either sandy loam (+) or red soil harvested leaves (**Figure 2ii**). However, the accumulation of cucurbitacin B appeared to be more dominant than cucurbitacin A.

**Figure 3:**
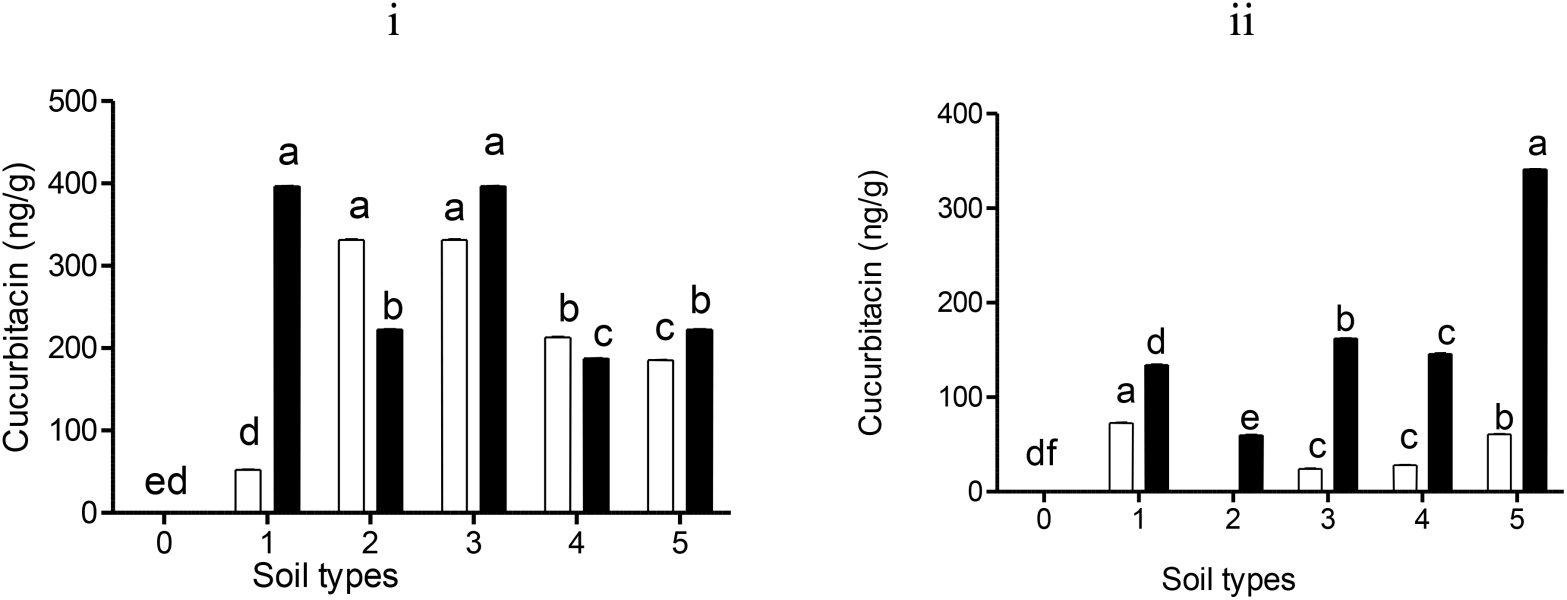
The accumulation of cucurbitacin A (□) and cucurbitacin B (▪) residues in tomato stems: (i) Nemarioc-AL and (ii) Nemafric-BL phytonematicide. The plants were grown under different soil types (0) sandy loam, (1) dark soil, (2) red soil, (3) silt soil, (4) sandy soil, and (5) sandy loam (+) (n = 36). Bars ( ± SE) with different letters showing significant differences between treatments at p ≤ 0.01.

In harvested stems from Nemarioc-AL phytonematicide-treated soils, the highest cucurbitacin A accumulation was found in both red soil and dark soil (331.6 ng/g), then sandy soil (213.2 ng/g), followed by sandy loam (+) (185.1 ng/g); dark soil showed the least (53 ng/g) (**Figure 3i**). However, the highest cucurbitacin B residues were observed in both red soil and silt soil (396.3 ng/g), followed by both dark soil and sandy loam (+) (222.5 ng/g); sandy soil showed the least (187.3 ng/g) (**Figure 3i**). In contrast, stems harvested from Nemafric-BL phytonematicide-treated soil types had the highest peak of cucurbitacin B residues in sandy loam (+) (341.1 ng/g), silt soil (162 ng/g), sandy soil (145.9 ng/g), and dark soil (134.3 ng/g); red soil showed the least (59.9 ng/g) (**Figure 3ii**). Cucurbitacin A accumulation in stems was detected in dark soil (72.5 ng/g), sandy loam (+) (60.8 ng/g), sandy soil (28.3 ng/g), and silt soil (24.3 ng/g); there were no traces of cucurbitacin A in red soil (**Figure 3ii**).

**Figure 4:**
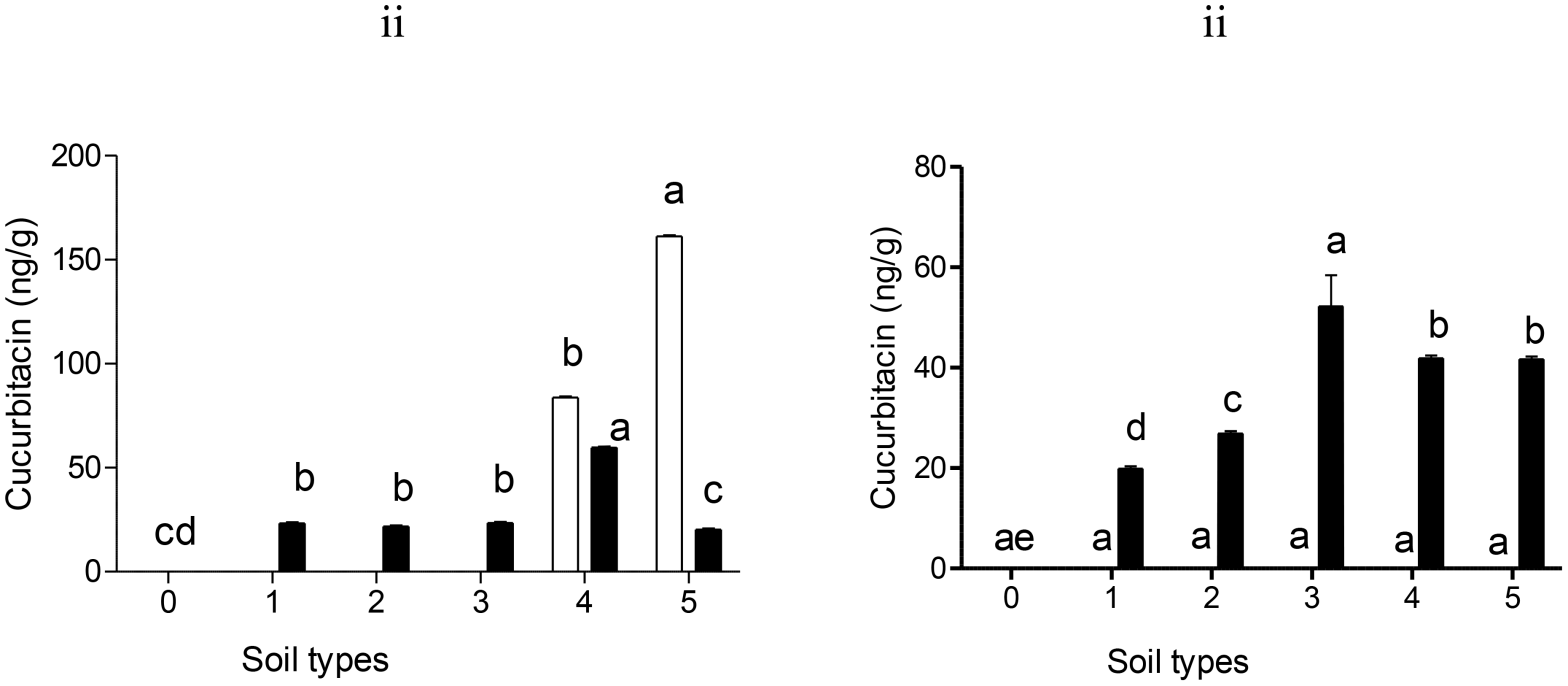
Levels of cucurbitacin A (□) and cucurbitacin B (▪) residues detected in tomato roots: (i) Nemarioc-AL and (ii) Nemafric-BL phytonematicide. The plants were grown under different soil types (0) sandy loam, (1) dark soil, (2) red soil, (3) silt soil, (4) sandy soil, and (5) sandy loam (+) (n = 36). Bars ( ± SE) with different letters showing significant differences between treatments at p ≤ 0.01.

In roots treated with Nemarioc-AL phytonematicide, the highest concentration observed was cucurbitacin A, with lower traces of cucurbitacin B (**Figure 4i**). In contrast, Nemafric-BL phytonematicide-treated roots had no traces of cucurbitacin A but predominantly possessed cucurbitacin B (**Figure 4ii**). In roots exposed to Nemarioc-AL phytonematicide on selected soil types, more cucurbitacin A residues were identified from sandy loam (+) (161.3 ng/g) to sandy soil (83.7 ng/g), and no residues were detected in dark soil, red soil, and silt soil (**Figure 4i**). Meanwhile, the levels of cucurbitacin B residues were as follows: sandy soil (59.5 ng/g), silt soil (23.4 ng/g), dark soil (23.3 ng/g), red soil (21.8 ng/g), and sandy loam (+) (20.3 ng/g) (**Figure 4i**). In contrast, in roots from Nemafric-BL phytonematicide-treated soil types, cucurbitacin A residues were not detected in all soil types (**Figure 4ii**). However, cucurbitacin B residues were detected in silt soil (52.23 ng/g), sandy soil (41.9 ng/g), sandy loam (+) (41.7 ng/g), and red soil (26.9 ng/g); dark soil showed the least (19.9 ng/g) (**Figure 4ii**).

**Figure 5:**
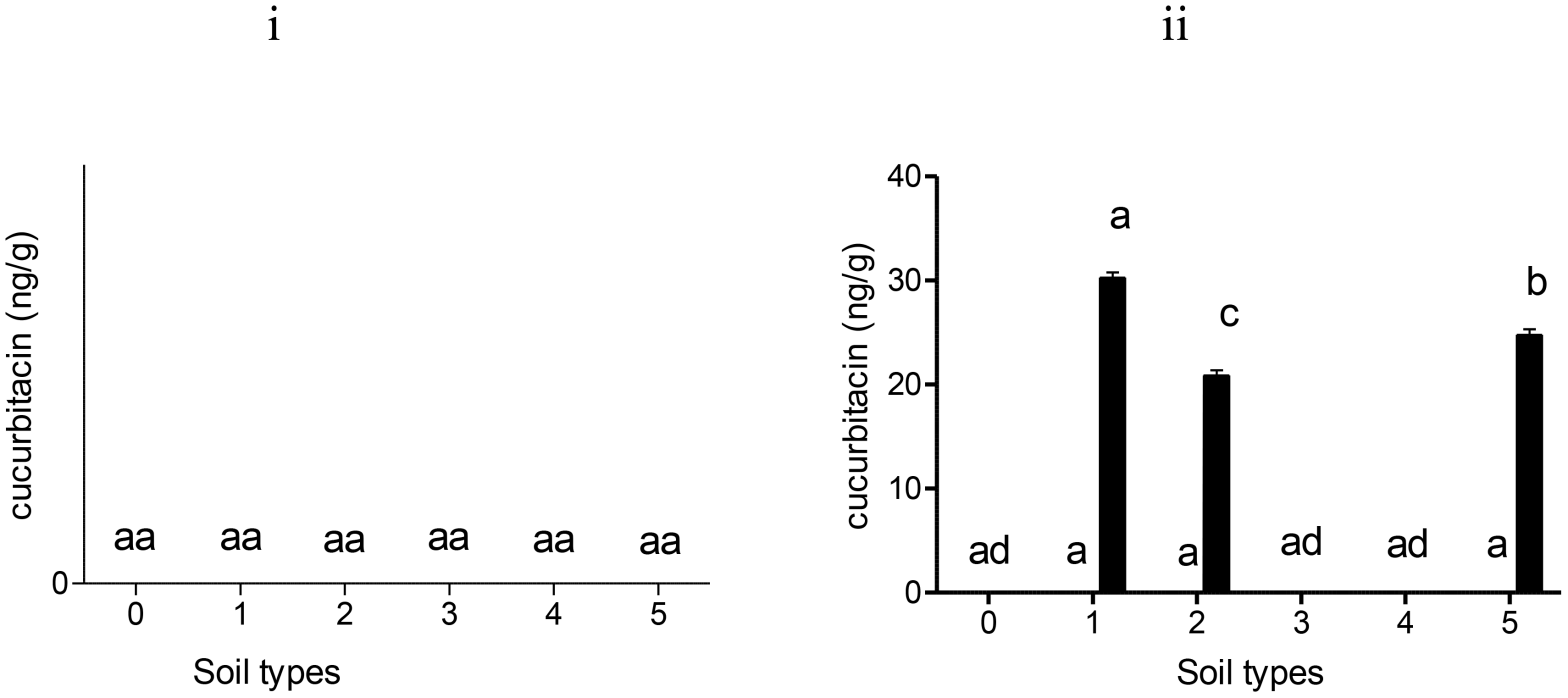
Levels of cucurbitacin A (□) and cucurbitacin B (▪) residues detected in soil treated with (i) Nemarioc-AL and (ii) Nemafric-BL phytonematicide. The different soil types were (0) sandy loam, (1) dark soil, (2) red soil, (3) silt soil, (4) sandy soil, and (5) sandy loam (+) (n = 36). Bars ( ± SE) with different letters showing significant differences between treatments at p ≤ 0.01.

The accumulation of cucurbitacin A and cucurbitacin B in sandy loam, dark soil, red soil, silt soil, sandy soil and sandy loam (+) treated by Nemarioc-AL phytonematicide was not detected (**Figure 5i**). Showing that Nemarioc-AL phytonematicide as a biopesticide is easily biodegradable in soil leaving no traces of cucurbitacin A and cucurbitacin B. In contrast, the cucurbitacin B residues were detected in dark soil, red soil and sandy loam (+) (**Figure 5ii**). The highest accumulation of cucurbitacin B was observed in dark soil (30.3 ng/g), after that sandy loam (+) (24.7 ng/g) and red soil (20.9 ng/g) was the least (**Figure 5ii**). No accumulation of cucurbitacin B residues was observed in silt and sandy loam soil types. The presence of cucurbitacin B on the stated soil types shows that this active ingredient is slowly biodegradable compared to cucurbitacin A without any traces in some soil types.

### 3.2 *In vitro* condition

#### 3.2.1 Viability profiles of the phytonematicides

**Figure 6:**
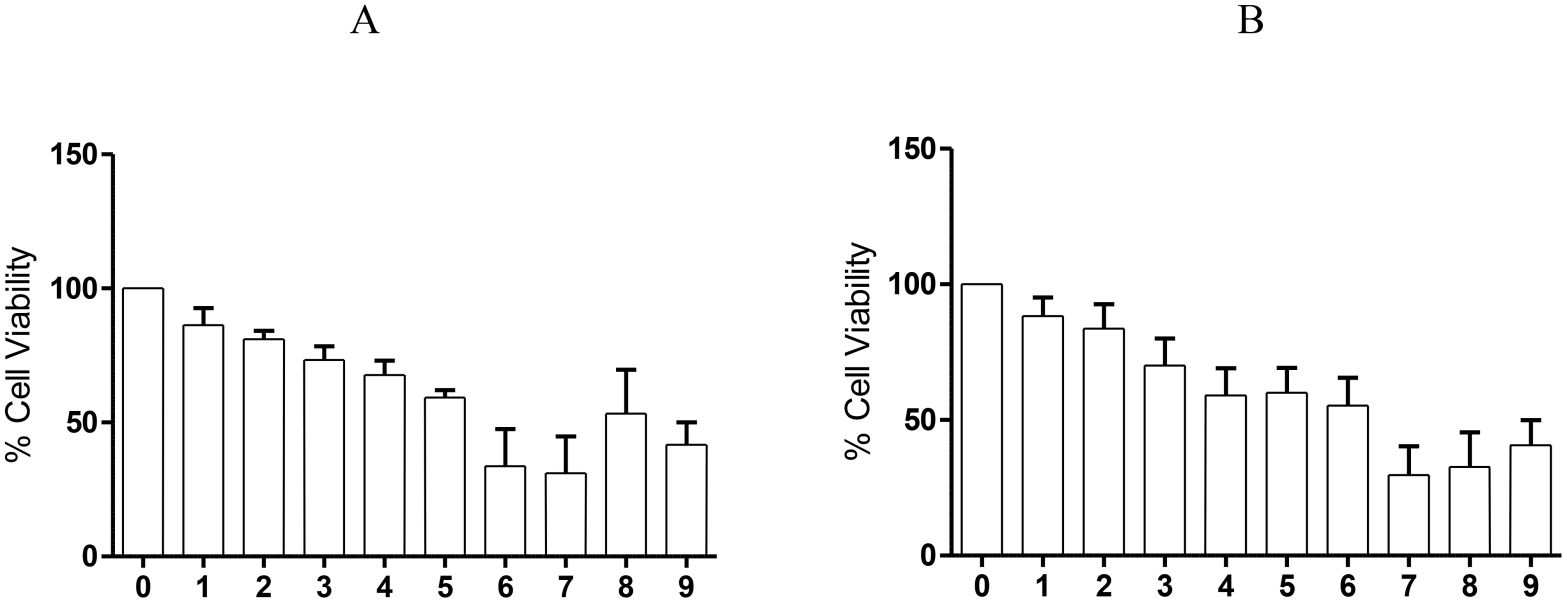
The response of murine Raw 264.7 macrophages to different concentrations [(1) 0.078, (2) 0.156, (3) 0.313, (4) 0.625, (5) 1.25, (6) 2.5, (7) 5, and (8) 10 mg/mL)] of Nemarioc-AL phytonematicide **(A)** and Nemafric-BL phytonematicide **(B)** under *in vitro* conditions. With (0) control and (9) control (+).

High concentrations of Nemarioc-AL phytonematicide (A) and Nemafric-BL phytonematicide (B) (2.5, 5 and 10 mg/mL) significantly reduced the viability of the Raw 264.7 cell line to below 50%, relative to the untreated control (**Figures 6A, B**). In contrast, low concentrations (1.25, 0.625, 0.313, 0.156, and 0.078 mg/mL) of Nemarioc-AL and Nemafric-BL phytonematicides resulted in more than 50% cell viability relative to the untreated control (**Figures 6A, B**).

**Figure 7:**
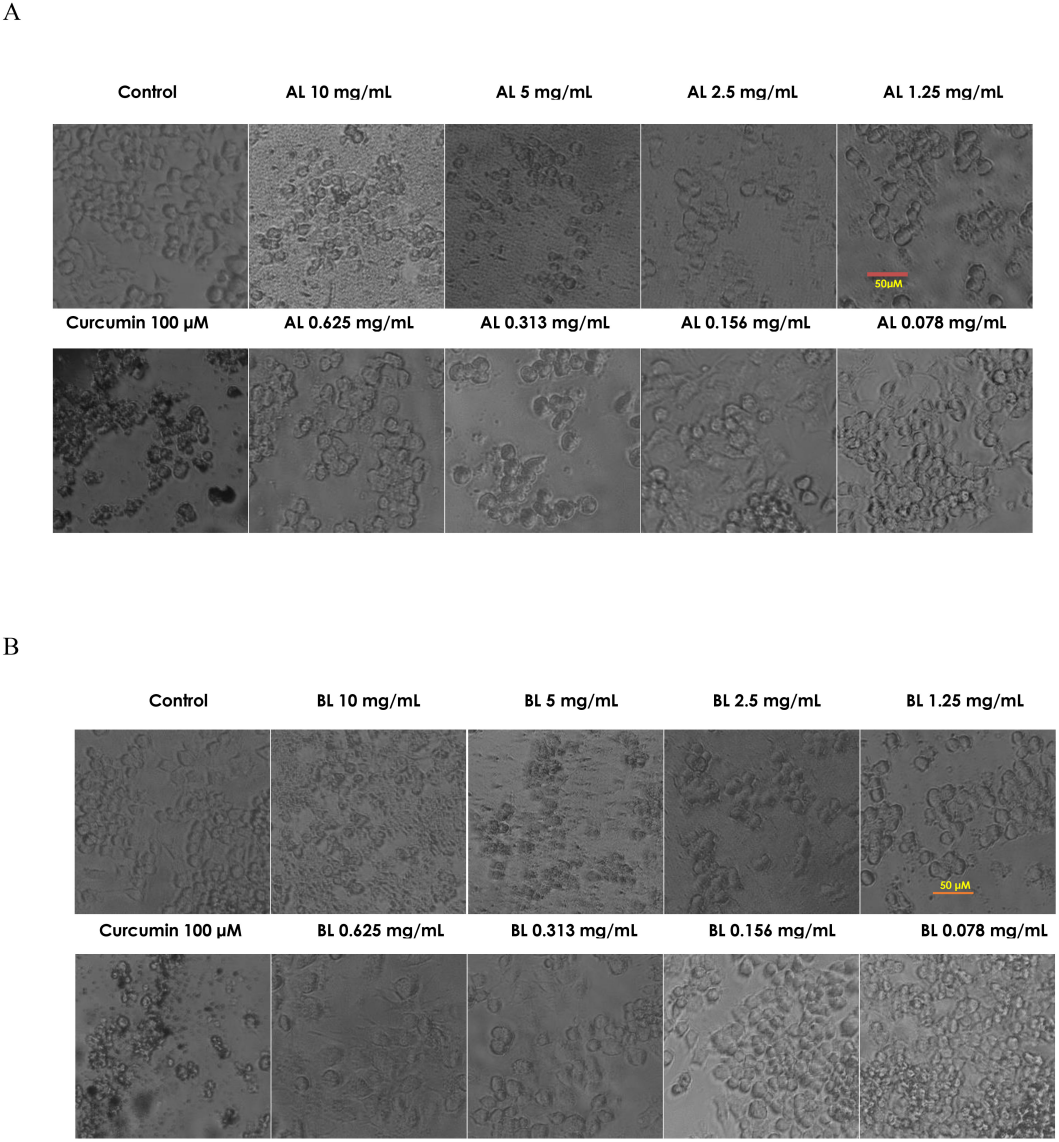
Cell morphology of murine Raw 264.7 macrophages to Nemarioc-AL phytonematicide **(A)** and Nemafric-BL phytonematicide **(B)** was captured using Olympus CKX33. LC micro software (Japan).

Raw 264.7 cells treated with Nemarioc-AL and Nemafric-BL phytonematicides exhibited altered morphology at higher concentrations compared to lower concentrations (**Figures 7A, B**). At elevated concentrations (2.5, 5, and 10 mg/mL), both phytonematicides induced signs of toxicity; cells lost their characteristic spindle shape and appeared shrunken (**Figures 7A, B**). Conversely, at concentrations ranging from 0.36 to 1.25 mg/mL, cell size increased, indicating reduced stress levels.

The IC_50_ values for Nemarioc-AL and Nemafric-BL phytonematicides on Raw 264.7 were established at 0.55 and 1.6 mg/mL, respectively (**Table 4**). Depicting negligible to moderate toxicity of the products. The lower the IC_50_ value, the more toxic the product; the higher the IC_50_ value, the safer the product. Therefore, Nemarioc-AL phytonematicide (IC_50_ = 0.55 mg/mL) was moderately toxic to the Raw 264.7 cell line, while Nemafric-BL phytonematicide (IC_50_ = 1.6 mg/mL) was non-toxic to the stated cell lines.

**Table 4:** Calculated IC_50_ of Nemarioc-AL and Nemafric-BL phytonematicides in mg/mL.

Product name	Active ingredient	IC_50_ value (mg/mL)
Nemarioc-AL phytonematicide	Cucurbitacin A	0.55
Nemafric-BL phytonematicide	Cucurbitacin B	1.06

#### 3.2.2 Apoptotic profiles of phytonematicides

**Figure 8:**
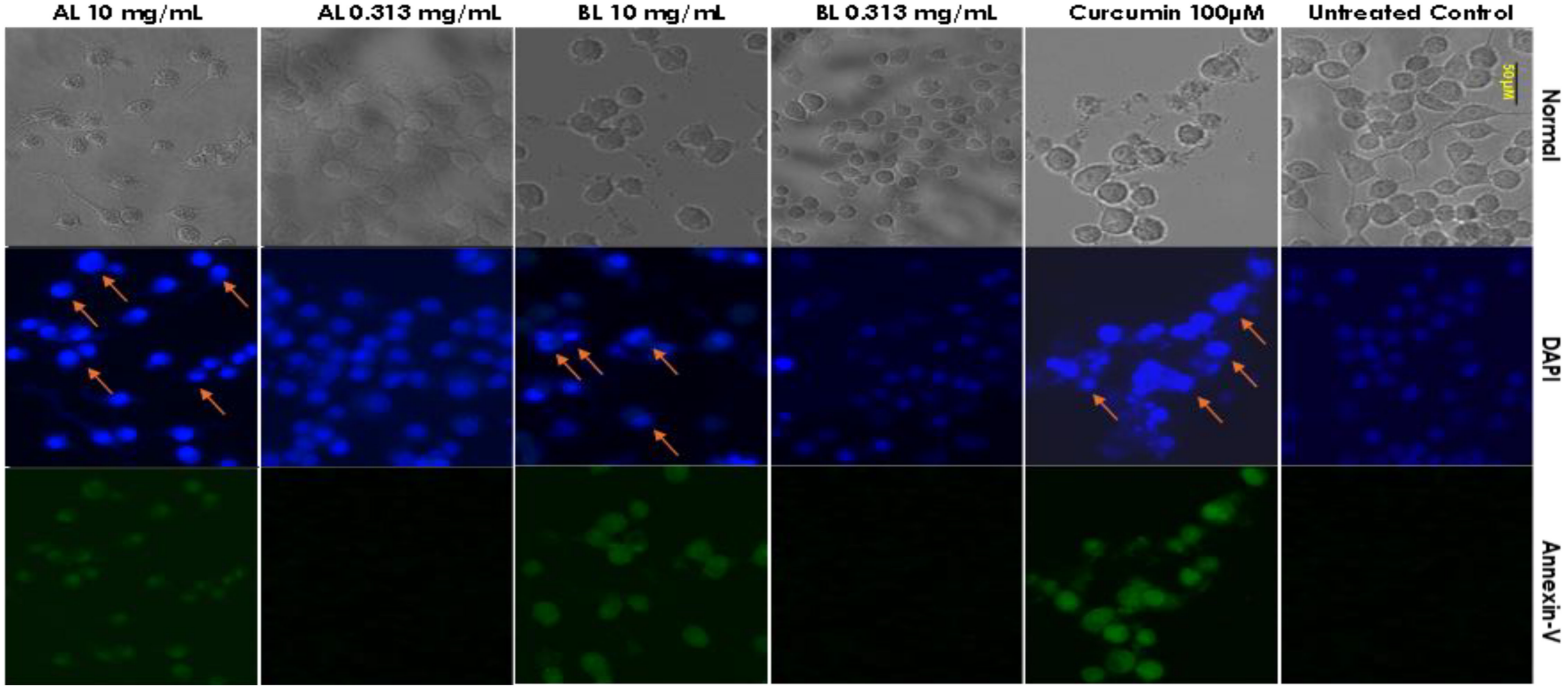
The evaluation of mechanism of cell death induced by high concentrations (0.313 and 10 mg/mL) of Nemarioc-AL and Nemafric-BL phytonematicides on murine Raw 264.7 macrophages. Untreated cells serve as control, and 100 µM curcumin is the (+) control.

Similarly, the phytonematicides Nemarioc-AL and Nemafric-BL at a low concentration of 0.313 mg/mL did not induce apoptosis, as the cells stained negative when treated with Annexin-V, showing no signs of chromosomal condensation, as illustrated with DAPI DNA staining (**Figure 8**). However, the high concentration (10 mg/mL) of both phytonematicides caused apoptosis as evident by positive staining when treated with Annexin-V, similar to the control curcumin applied to the cells at 100 μM. They also showed chromosomal condensation, as DAPI staining was intense at the high concentrations of phytonematicides as compared to the untreated control (**Figure 8**), with chromatin condensation in cells treated with 10 mg/mL of both phytonematicides, similar to the positive control (curcumin at 100 μM), indicated by arrows. This phenomenon illustrates membrane flipping, one of the early features of apoptosis.

## 4 Discussion

### 4.1 Under greenhouse conditions

Nemarioc-AL and Nemafric-BL phytonematicides decreased the pH levels of all selected soil types. A reduction of pH in soil types could be equivalent to the actual pH of phytonematicides, which is 3.7 (Mashela et al., 2017). As a result, long-term use of the phytonematicides will drastically reduce the soil pH to an acidic level, as observed in the current study. Once a soil type becomes acidic, it will interrupt the normal chemical properties of the soil, resulting in the dissolution of some chemical compounds for plant uptake. This will promote the accumulation of residues in plant parts, which is a serious constraint for crop production (Hue, 2011). Acidic soil resulting from phytonematicides can negatively impact macro- or microorganisms and decrease the degradation of toxic substances, leading to prolonged bioavailability in the soil (Golla, 2019).

In the current study, Nemarioc-AL phytonematicide increased soil electrical conductivity on the stated soil types. These results corroborate findings by Mashela (2002), where Nemarioc-AG phytonematicide increased electrical conductivity levels. An increase in electrical conductivity is generally undesirable, as it depicts higher salinity and plant stress. Hence, it increases the chances of accumulation of residues in produce. This contradicts findings on tomato plants grown in a hydroponic system, where high concentrations of electrical conductivity increased growth and yield (Rosadi et al., 2014). In contrast, Nemafric-BL phytonematicide in the current research had increased, decreased, or had no effects on soil electrical conductivity. A decrease in soil electrical conductivity is usually ideal to avoid salinity. Soil electrical conductivity was maintained in the untreated control and red soil for desirable crop growth. Despite trends observed in soil electrochemical conductivity before and after planting in the current study, most of the observed electrical conductivity was increased by the phytonematicides. This could be that the soil has already had an accumulation of soluble salts like potassium, nitrogen, and phosphate prior to its use in this study. Additionally, all the soil types were collected from agricultural areas where synthetic chemical fertilizers are used on a daily basis.

Plant protection depends on the use of synthetic chemical pesticides and biopesticides to enhance production (Aguilar-Marcelino et al., 2023). After the yield and quality of produce are attained, information on the presence of chemical residues in produce is usually ignored. However, the presence of chemical residues in food can pose serious side effects on human health. In the current study, the presence of chemical residues in fruits treated with phytonematicides was detected. These results confirm findings where neem (*Azadirachta indica* Juss.) essential oil was used as a bioinsecticide against sucking insect pests on eggplant (*Solanum melongena* L.) production; azadirachtin, the active ingredient in neem, was also detected in fruits (Takla et al., 2021). In contrast, the results contradict the finding that Nemarioc-AL and Nemafric-BL phytonematicides resulted in no accumulation of chemical residues in tomato fruits (Shadung et al., 2017; Bango, 2019). Also, the presence of cucurbitacin residues was not detected on indigenous green leafy vegetable nightshade (*Solanum retroflexum* Dun.) treated with phytonematicide (Malebe, 2019). Currently, advanced technological systems and modern machines have made it easier to detect the presence of cucurbitacin residues in produce. In the current study, fruits treated with Nemarioc-AL phytonematicide only accumulated cucurbitacin B as opposed to cucurbitacin A. However, cucurbitacin A was not detected in fruits treated with Nemafric-BL phytonematicide. As stated, the bioactive compound of Nemarioc-AL phytonematicide is cucurbitacin A (C_32_H_46_O_9_), while Nemafric-BL phytonematicide possesses cucurbitacin B (C_32_H_46_O_8_). The non-detection of cucurbitacin A in tomato fruits treated with Nemarioc-AL phytonematicides shows that it is probable that the bioactive compound of Nemarioc-AL phytonematicide is transformed into cucurbitacin B by plant enzymes. The non-detection of cucurbitacin A in fruits harvested from Nemafric-BL-treated soil types indicates that cucurbitacin A was below a traceable level and not detected during analysis.

Cucurbitacin A and cucurbitacin B, both bioactive compounds, were detected in leaves harvested from phytonematicide-treated soil types, with cucurbitacin B being more dominant than cucurbitacin A. This finding illustrates that more than one cucurbitacin was isolated from *C. myriocarpus* and *C. africanus* fruits and corroborates findings where cucurbitacin A and cucurbitacin C were isolated in leaves of *C. myriocarpus* (Shaik et al., 2017). These confirm why Nemarioc-AL and Nemafric-BL phytonematicides as biopesticides possess multiple modes of action (Mashela et al., 2015; Dube, 2016; Fenibo et al., 2021). In stems, cucurbitacin B was also dominant compared with cucurbitacin A. This illustrates that cucurbitacin B is a non-polar chemical compound that is highly stable and not easily degradable as compared to cucurbitacin A, which partially dissolves in water (Chen et al., 2005). In roots, high concentrations of cucurbitacin A in roots harvested from Nemarioc-AL phytonematicide-treated soil types confirm that cucurbitacin A is more abundant in *C. myriocarpus* than cucurbitacin B. A non-detectable cucurbitacin A residue in tomato roots harvested from Nemafric-BL phytonematicide-treated soil types shows that cucurbitacin B is abundant in *C. africanus*. Lastly, Nemarioc-AL phytonematicide-treated soil types had no accumulation of cucurbitacin residues. In contrast, Nemafric-BL phytonematicide-treated soil types had cucurbitacin B residues without traces of cucurbitacin A. The results confirm findings by Bango (2019), where the stated phytonematicides did not leave residues in soil. This could be because Nemarioc-AL phytonematicide is a biopesticide partially soluble in water and easily biodegradable, as compared to Nemafric-BL phytonematicide, which is non-polar and takes more time to degrade in the soil. Just like other biopesticides, if applied in the soil, they easily become biodegradable, leaving no traces as compared to many synthetic chemical nematicides with persistent chemical residues (Soyel et al., 2023).

### 4.2 *In vitro* conditions

Although the complements of Nemarioc-AL and Nemafric-BL phytonematicides are effective against nematodes and environmentally friendly, improper handling during application or residue accumulation in produce can result in human exposure. This concept of safety on biopesticides has always been disregarded, but the reality was that it always occurred unintentionally. Therefore, exposure to these foreign bioactive compounds, technically called xenobiotic chemicals (León-Mejía et al., 2021), can be toxic to humans, especially those developed as biopesticides. Assessing the cytotoxicity level of the stated phytonematicides in question is prudent. The response of living entities to Nemarioc-AL and Nemafric-BL phytonematicides is described in a density-dependent growth (DDG) pattern (Mashela et al., 2017). This pattern was conceptualized into stimulation, neutral, and inhibition phases. At the stimulation phase, which is a low concentration of phytonematicides, the products were non-toxic to Raw 264.7 cell lines. In the neutral phase, neither stimulation nor inhibition of cells occurred. The inhibition phase showed that the products were cytotoxic to cell lines, and this occurred at high concentration levels. This pattern was observed when Nemarioc-AL and Nemafric-BL were used to manage plant parasitic nematodes in plants (Bango, 2019; Malebe, 2019). The observed results agree with the findings that different plant extracts from *Acmella ciliata* (Kunth) Cass., *Coriandrum sativum* L., and *Glebionis coronaria* L. at higher concentration levels (320 to 640 μg/mL) induced mortality on Vero cells (Chan et al., 2015), hence resulting in reduced cell viability. In contrast, lower concentration levels of phytonematicides on Raw 264.7 cells affected viability, with an acceptable percentage of more than 50% showing moderate to no cytotoxicity of the product. These confirm the previous publication that plant extracts from *Andrographis paniculata* (Burm.f.) Wall. ex Nees and *A. paniculata* over time (24 to 48 hrs) at different concentration levels (4–500 μg/mL) were not cytotoxic to murine hepatocyte, thymocyte, and splenocyte cell lines (Ala et al., 2018). Hence, cells were more viable than at higher concentration levels, suggesting a dose-dependent response.

Cell morphology serves as a key indicator for detecting cell stress, which is often associated with toxicity. Toxicity was observed on Raw 264.7 cells treated with Nemarioc-AL and Nemafric-BL phytonematicides. Cells exhibited altered morphology at higher concentration levels, with cells losing their characteristic spindle shape and appearing shrunken. This morphological change suggests that the cells experienced substantial stress, ultimately leading to cell death (Istifli et al., 2019). Conversely, at the lower concentration range, cell size increased, indicating reduced stress levels. The observed increase in cell size at lower doses may suggest cellular growth, as cells typically enlarge prior to mitosis and division (Shkolyar et al., 2015). At low concentration, cells regained their normal spindle shape and returned to their typical size.

Necrotic cell death, unlike apoptosis, can lead to severe complications due to its pro-inflammatory nature. In contrast, apoptosis is a programmed form of cell death that occurs in a controlled manner without triggering inflammation or its associated complications. Chromatin condensation occurred in cells treated with both phytonematicide and positive control, describing an inhibition phase or toxicity of the products. Additionally, the cells stained positive for Annexin-V, indicating that the phytonematicides induce apoptosis. Although chromatin condensation is a normal event during mitosis, when it occurs as a result of apoptosis, it activates the DNA damage response system, which subsequently leads to other apoptotic features and the formation of apoptotic bodies (Burgess et al., 2014). Therefore, these phytonematicides, at high concentrations, induce apoptosis, while at lower concentrations, they appear to be safe for use.

## 5 Conclusion

Cucurbitacins A and B were present in both phytonematicides. Residue accumulation in tomato plant parts was independent of the treated soil type and the type of phytonematicide applied to the soil. The response of Raw 264.7 cell lines to phytonematicides was characterized in a DDG pattern with a non-toxic stimulation phase and a toxic inhibition phase. The products showed negligible to moderate toxicity, supporting their potential as safe phytonematicides. Furthermore, their demonstrated safety on the RAW 264.7 cell line indicates minimal risk to non-target mammalian systems, supporting their broader safety profile. Phytonematicides were shown to be safe to the Raw 264.7 cell line, suggesting that they are suitable for their intended use in managing plant parasitic nematodes. It is recommended that farmers use the non-toxic doses established for the safety of both products. The study is limited to tomato plants, selected soil types, targeted metabolites, greenhouse conditions, and a single cell line (Raw 264.7). These limitations mean that the results may not entirely reflect field conditions, different crops or soil types, or *in vivo* responses. Future research is suggested to evaluate residue accumulation in green leafy vegetables, examine non-targeted metabolites, test cytotoxicity in additional mammalian cell lines, and assess long-term environmental impacts of phytonematicides on orchards.

## Data Availability

The raw data supporting the conclusions of this article will be made available by the authors, without undue reservation.
